# Acanthosis nigricans du visage révélant un adénocarcinome bronchique primitif: à propos d’un cas

**DOI:** 10.11604/pamj.2021.39.250.30524

**Published:** 2021-08-18

**Authors:** Youssef Bougrini, Reda Belghol, Younes Elkhachine, Hicham Naji-Amrani, Aziz Ouarssani

**Affiliations:** 1 Service de Pneumo-phtisiologie, Hôpital Militaire Moulay Ismail, Meknès, Maroc,; 2 Service de Dermatologie, Hôpital Militaire Moulay Ismail, Meknès, Maroc,; 3 Faculté de Médecine et de Pharmacie, Université Sidi Mohammed Ben Abdellah, Fès, Maroc

**Keywords:** Acanthosis nigrigans, adénocarcinome bronchique, à propos d’un cas, Acanthosis nigrigans, bronchial adenocarcinoma, case report

## Abstract

Nous rapportons le cas d'un cancer bronchique révélé par un acanthosis nigrigans du visage. Ce mode de révélation qui est rare, peut précéder de plusieurs mois le diagnostic de néoplasie sous-jacente. Cette observation souligne l'intérêt de rechercher un cancer primitif pulmonaire en cas d´acanthosis nigrigans.

## Abstract (en)

We here report a case of bronchial cancer revealed by acanthosis nigrigans affecting the face. This rare mode of revelation may precede diagnosis of underlying neoplasia by several months . This study highlights the importance of suspecting primary lung cancer in patients with acanthosis nigrigans.

## Introduction

L'acanthosis nigrigans (AN) est une dermatose rare qui est souvent associée à des affections bégnines, principalement au diabète par insulino-résistance. Dans des cas exceptionnels, il peut accompagner une néoplasie profonde dont l´identification s'avère parfois difficile. Nous rapportons dans cette observation le cas rare d'un adénocarcinome bronchique métastatique révélé par un AN du visage.

## Patient et observation

**Informations du patient:** il s´agit d´un patient âgé de 58 ans, tabagique chronique à raison de 40 paquets années, a consulté en dermatologie pour l´apparition de façon symétrique au niveau des deux pommettes de plaques irrégulières, pigmentées, verruqueuses, de couleur brunâtre devenant de plus en plus sombre, indolores et augmentant progressivement de taille. Un bilan biologique demandé était normal à savoir une glycémie à jeun et une cortisolémie de 8h00. Le patient a été mis sous traitement symptomatique, une crème à base de vitamine A à été prescrite. Trois mois plus tard, l´évolution a été marquée par l´accentuation des lésions dermatologiques du visage et l´installation d´une toux productive avec hémoptysie de faible abondance, associée à des névralgies cervico-brachiales droites, évoluant dans un contexte de fléchissement de l´état général.

**Figure 1:**
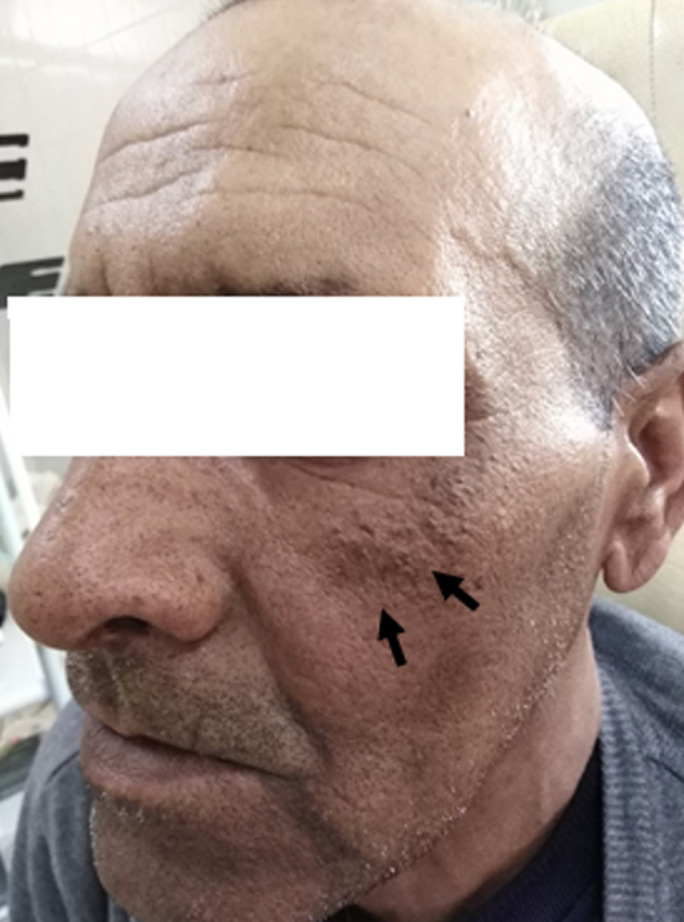
acanthosis nigricans du visage (flèches)

**Figure 2:**
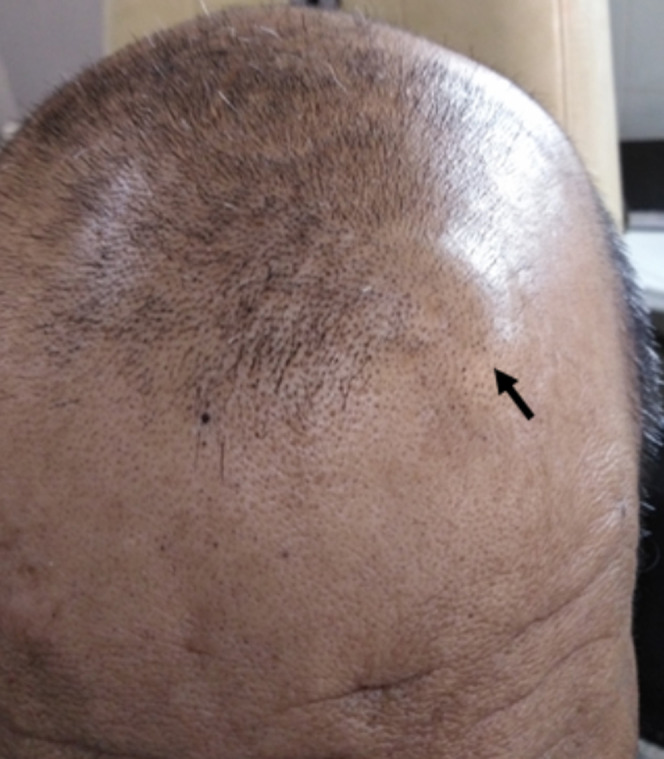
nodule sous-cutané du cuir chevelu

**Résultats cliniques:** l'examen clinique pleuropulmonaire a objectivé un syndrome de condensation apical de l´hémithorax droit. L'examen cutanéo-muqueux a montré des plaques pigmentées, brunes, épaisses et papillomateuses (Figure 1), mesurant 4 à 5 cm de grand diamètre et intéressant de façon symétrique les régions sous-orbitaires sans d´autres localisations faisant évoquer un acanthosis nigricans. L´examen du cuir chevelu a noté la présence d´un nodule sous cutané de 2cm de diamètre, de caractère ferme, indolore et mobile siégeant dans la région pariétale droite (Figure 2).

**Figure 3:**
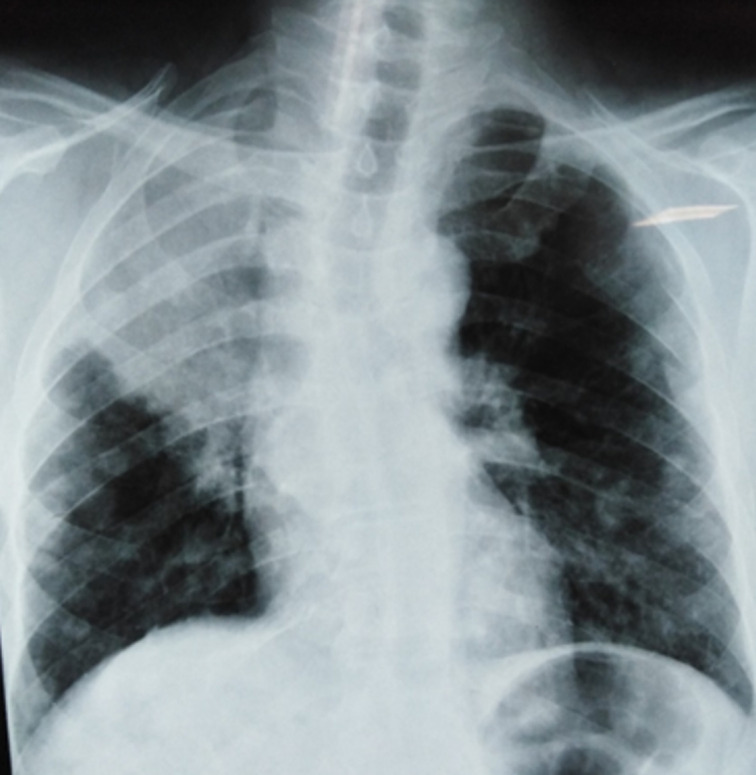
radiographie thoracique de face opacité pulmonaire droite avec des nodules bilatéraux

**Figure 4:**
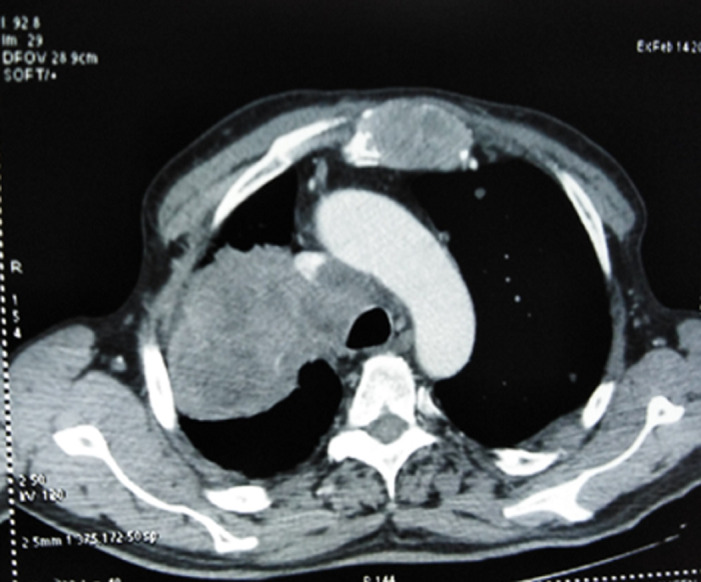
tomodensitometrie (TDM) thoracique processus tumoral hilo-apical droit

**Figure 5:**
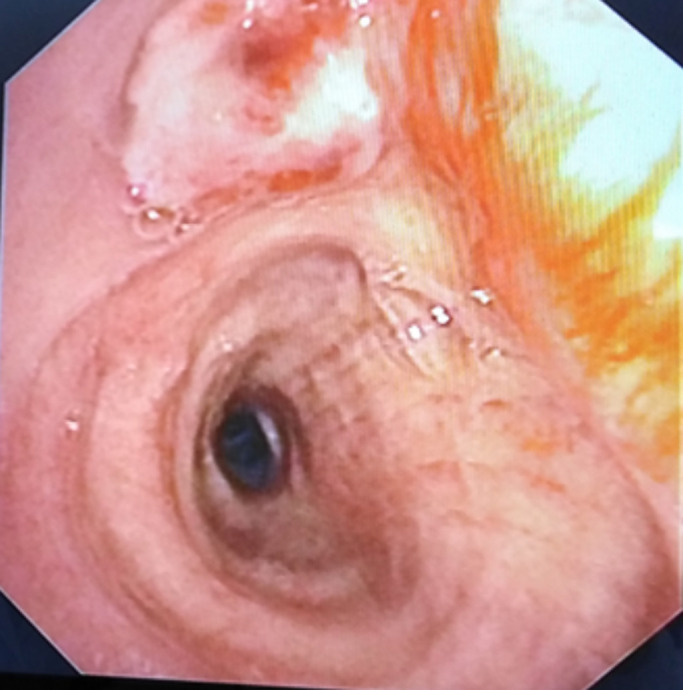
fibroscopie bronchique montrant une sténose bourgeonnante de la bronche lobaire supérieure droite

**Démarche diagnostique:** la radiographie thoracique de face a objectivé une opacité hétérogène et rétractile prenant la moitié supérieure du champ pulmonaire droit, associée à des opacités nodulaires et macronodulaires diffuses bilatérales en lâcher de ballons (Figure 3). La TDM thoracique a mis en évidence une masse tissulaire apico-hilaire droite, à centre nécrosé, des limites irrégulieres, avec une adénopathie sous-carinaire (Figure 4). La fibroscopie bronchique a objectivé un bourgeon tumoral blanchâtre obstruant complétement l'orifice de la bronche lobaire supérieure droite (Figure 5). Des biopsies ont été faites, l´étude anatomopathologique et l´immunohistochimie ont confirmé le diagnostic de l'adénocarcinome bronchique primitif. La biopsie du nodule sous-cutané a été faite et l'histologie a confirmé qu´il s´agit d'une métastase cutanée d´un adénocarcinome bronchique. Un bilan d'extension a été fait: le scanner thoraco-abdomino-pelvien a mis en évidence une métastase surrénalienne avec un lâcher de ballon pulmonaire bilatéral.

**Intervention thérapeutique et suivi:** la décision thérapeutique après la réunion de concertation pluridisciplinaire était de commencer une chimiothérapie exclusive.

## Discussion

Les syndromes paranéoplasiques sont des perturbations physiopathologiques qui affectent environ 8% des patients atteints de cancer [1]. Elles sont souvent décelées après le diagnostic de la néoplasie primitive. Cependant, leur découverte peut être inaugurale et permettra le diagnostic à un stade plus précoce. L´AN a été décrit pour la première fois par Pollitzer et Janvosky [2] comme étant une dermatose caractérisée par un épaississement cutané symétrique, de pigmentation brune et à surface veloutée ou verruqueuse, localisées préférentiellement dans les grands plis de flexion notamment les aisselles, les plis inguinaux et la nuque, et dans les régions latéro-cervicales. L´atteinte du visage constitue une localisation exceptionnelle, à noter qu'il existerait des formes diffuses. Au microscope, l'aspect de l´acanthosis nigrigans est identique quelle que soit sa localisation : il s´agit d´une acanthose avec hyperkératose et papillomatose. L'hyperkératose s'invagine très largement dans les dépressions de l´épiderme, expliquant l'aspect anfractueux des lésions. On trouve en outre une hyperpigmentation continue de la basale sans augmentation du nombre des mélanocytes [3]. L'aspect est proche des kératoses séborrhéiques ou des nævus épidermiques verruqueux.

Plusieurs descriptions dans la littérature ont été rapporté, associant AN à de nombreuses affections cliniques, liées dans la plupart des cas à des entités insulinorésistantes [4], telles que l´obésité, le diabète de type 2, le syndrome métabolique, le syndrome des ovaires polykystiques, et d´autres endocrinopathies à savoir le syndrome de Cushing et hypothyroïdie... Parfois, l´acanthosis nigricans est associé à des syndromes génétiques et à la consommation de certaines drogues comme acide nicotinique et acide fusidique, mais aussi les hormones) [5]. En 1893, Ferdinand-Jean Darier a décrit pour la première fois l'association d'AN avec les tumeurs malignes [6], qui, bien que cliniquement très semblable, touche généralement les paumes et les plantes, d´apparition et d´installation plus rapide, affectant beaucoup plus les sujets âgés.

L´AN malin peut soit précéder (18%), accompagner (61%) ou suivre (21%) l´apparition d´une néoplasie interne [7]. La pathogénie est liée à la sécrétion inappropriée par la tumeur de facteurs de croissance (EGF, TGFa) ou d´anticorps anti-récepteurs à l´insuline. Le TGFa produit par la tumeur favorise la prolifération des cellules épidermiques (mécanisme endocrine). Le TGFa et l´EGF ont des analogies structurelles et exercent leur action sur le même récepteur situé à la surface des cibles cellulaires [8]. L´acanthosis nigricans d´origine maligne est communément associé à des cancers intra-abdominaux (94%) [9], le cancer de l´estomac vient en première position, suivi de celui du pancréas représentant 61 à 69% de l´association cancer et acanthosis nigricans [10]. Le type histologique le plus fréquemment retrouvé de cette association est l´adénocarcinome, néanmoins plusieurs auteurs ont rapporté d´autres cas histologiques à type de sarcomes, de schwannomes, de leucémies, de cancers à cellules transitionnelles du rein, de tumeurs de Wilms.

La coexistence de l´AN et du cancer bronchique est rare, le premier cas a été rapporté en 1914 [11]. Curth *et al*. [12] ont largement examiné l'association des tumeurs pulmonaires et d’AN, concluant qu´il s´agit le plus souvent d´un adénocarcinome bronchique diagnostiqué au stade métastatique. Dans ce cas, l´AN touche des zones atypiques (la cavité buccale, le visage, les mains, les pieds), et augmente l´intensité rapidement. Le prurit généralisé ou localisé affectant le site des papillomes, est également un signe important pouvant être associé à un stade tardif. Le traitement d´AN est celui de la malignité sous-jacente. L´importance des lésions évolue parallèlement à l´évolution de la maladie cancéreuse. Les lésions cutanées tendent à disparaître ou à diminuer avec le traitement de la cause maligne responsable. Cependant, pour certaines pathologies ayant reçu une chimiothérapie, la réduction ou la disparition de AN peut être due soit à la diminution de la production tumorale des facteurs de croissance, soit à l´effet de la chimiothérapie elle-même; néanmoins, la réapparition de AN reste un bon signal d´alarme de la progression tumorale.

## Conclusion

La néoplasie constitue l´origine de l´acanthosis nigricans dans 20% des cas, et dont il peut être le mode révélateur. Par conséquent, si un patient accuse une apparition soudaine de l'AN, nous devons faire une anamnèse et un examen clinique minutieux à la recherche d´une tumeur maligne sous-jacente.
